# Predictive Modeling of Heart Failure Outcomes Using ECG Monitoring Indicators and Machine Learning

**DOI:** 10.1111/anec.70097

**Published:** 2025-06-27

**Authors:** Jia Liu, Dan Zhu, Lingzhi Deng, Xiaoliang Chen

**Affiliations:** ^1^ Department of Cardiology, Zone 6 The First People's Hospital of Chenzhou Chenzhou HuNan China

**Keywords:** electrocardiographic monitoring, heart failure, machine learning, predictive modeling, random forests, SHAP interpretation

## Abstract

**Background:**

Heart failure (HF) is a major driver of global morbidity and mortality. Early identification of patients at risk remains challenging due to complex, multivariate clinical relationships. Machine learning (ML) methods offer promise for more accurate prognostication.

**Objective:**

We evaluated the predictive value of electrocardiogram (ECG)–derived features and developed an ML model to stratify HF risk.

**Methods:**

We analyzed a public cohort of 1061 patients, of whom 589 (55.5%) developed HF. Records were randomly divided into training (70%, *n* = 742) and test (30%, *n* = 319) sets. After preprocessing, we trained a random forest (RF) classifier. Performance on the test set was assessed via accuracy, sensitivity, specificity, F1 score, and area under the receiver operating characteristic curve (AUC). Feature selection employed Gini importance and the Boruta algorithm, while SHAP values provided model interpretability.

**Results:**

The RF model achieved an AUC of 0.969, with 91.8% accuracy, 93.8% sensitivity, 89.4% specificity, and a 92.7% F1‐score. The top predictors included ST depression (Oldpeak), maximum heart rate (MaxHR), ST‐segment slope, and serum cholesterol. Confusion matrix analysis confirmed robust discrimination between HF and non‐HF cases. SHAP interpretation reinforced the dominant influence of ECG‐related indices and cholesterol on individual risk estimates.

**Conclusion:**

An RF model leveraging ECG features demonstrated excellent performance for HF risk prediction and highlighted key physiologic markers. Future work should integrate comorbidity profiles and detailed biochemical data to further enhance clinical applicability.

## Graphical abstract

This study developed a machine learning model using ECG and clinical data to accurately predict heart failure. Key features such as ST‐Slope, MaxHR, and Oldpeak contributed to the model's high performance (AUC = 0.969), supporting early risk identification and clinical decision‐making.

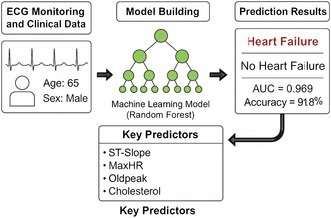

## 1 Introduction

Heart failure (HF) is a complex clinical syndrome responsible for over one‐third of cardiovascular‐related deaths worldwide (James et al. 2018). It affects millions globally, significantly diminishing patients' quality of life, increasing hospitalizations and healthcare costs, and placing a heavy burden on health systems (Azzopardi et al. 2023). Asia is the fastest growing and most populous region in the world, accounting for 60% of the global cardiovascular disease burden (Yoo et al. 2024).

Early diagnosis and prediction of HF are critical for optimizing treatment options, slowing disease progression, and reducing mortality. Traditionally, HF diagnosis relies on clinical symptoms and signs supported by ancillary tests such as B‐type natriuretic peptide (BNP) levels and echocardiography (Januzzi et al. 2011). However, these methods have limitations: BNP can be affected by various factors, and echocardiography, while informative, is operator‐dependent and not universally available in resource‐limited settings.

The electrocardiogram (ECG) is a widely utilized, non‐invasive, real‐time, and cost‐effective tool for cardiovascular monitoring and diagnosis (Dickstein et al. 2008). ECG can capture changes in cardiac electrical activity (heart rate, rhythm disturbances, signs of myocardial ischemia) that are valuable for assessing cardiac function. Notably, ECG findings have been used to predict mortality in patients with acute HF (Václavík et al. 2014). However, traditional ECG analysis relies on manual interpretation by clinicians, which is subjective, time‐consuming, and difficult to scale for large populations.

In recent years, the advent of big data and improved computing power has spurred the application of machine learning (ML) in medical data analysis. ML algorithms can automatically extract patterns from complex, multi‐dimensional clinical data, build efficient predictive models, and assist in early diagnosis and prognostication. Studies have shown that ML performs well in cardiovascular disease prediction, for example in forecasting outcomes in HF using algorithms such as random forest (RF), support vector machines (SVM), and neural networks (Sabouri et al. 2023; Li et al. 2022). Nevertheless, research specifically on HF prediction using ECG indicators remains relatively limited. Existing studies often focus on a single or a few ECG metrics, neglecting the value of a comprehensive multivariate approach. Moreover, model interpretability and clinical utility are pressing concerns—achieving high predictive performance is important, but clinicians also need clear, interpretable rationale from these models for decision‐making.

In light of the above, this study aimed to develop an efficient and interpretable prediction model for HF using a broad set of ECG monitoring indicators and ML methods. We formulated the hypothesis that integrating multiple ECG‐derived metrics into a ML model would yield high accuracy in predicting HF outcomes. Specifically, we investigated whether a RF model using comprehensive ECG features could effectively stratify patients by HF risk and identify the key ECG predictors of HF. By doing so, we seek to provide clinicians with a reliable decision‐support tool to promote early diagnosis and individualized treatment of HF, ultimately improving patient outcomes.

## 2 Methods

### 2.1 Data Sources

The dataset for this study was sourced from the publicly available Kaggle ML Repository (Rahulraj, n.d.). The dataset contains 12 clinical and cardiac monitoring metrics from 1061 patients and aims to predict whether a patient is experiencing HF.

### 2.2 Ethical Considerations

The dataset used in this study is publicly available and contains only de‐identified data; no personal identifiers are present. As this study is a secondary analysis of an anonymized public dataset, it did not involve any direct patient contact or intervention. Thus, no institutional ethics approval was required for this analysis. The use of the data comply with relevant ethical guidelines and data usage policies associated with the source.

### 2.3 Study Population

All patients in the dataset (*N* = 1061) were included in this study, as the dataset was already defined as a cohort for HF prediction. This population included 637 men (60%) and 424 women (40%), all of whom had measurements for the specified 12 indicators.

Inclusion criteria: patients with complete data available for all the ECG and clinical features listed above. Since the dataset itself is a curated collection of heart disease patient records, we did not apply additional inclusion criteria such as age limits or disease severity—effectively, any record present in the dataset was considered if it contained the required fields.

Exclusion criteria: no patients were prospectively excluded beyond the absence of required data.

### 2.4 Data Preprocessing

The dataset was stratified and split into training and test sets at a 7:3 ratio, preserving the 55% HF prevalence in both. Preprocessing was performed post‐split to prevent test set information leakage, with parameters fitted on the training set and applied to the test set.

Missing Data: Cholesterol values of 0 mg/dL, deemed implausible, were imputed with the training set median to maintain sample size and distribution. Other continuous features had no significant missing data, and binary/categorical features had no missing entries.

Categorical Encoding: Binary features (ExerciseAngina, FastingBS) were encoded as 0/1. Multi‐category features (ChestPainType [ATA, NAP, ASY, TA], RestingECG [Normal, ST, LVH], ST_Slope [Up, Flat, Down]) were one‐hot encoded to create binary dummy variables, avoiding ordinal assumptions.

Feature Scaling: Continuous features (age, resting blood pressure, cholesterol, MaxHR, Oldpeak) were standardized to mean 0 and standard deviation (SD) 1 using Z‐score normalization, with training set parameters applied to the test set to ensure consistent scaling.

Data Splitting: Stratification by HF outcome ensured proportional class distribution. The training set was used for model development, while the test set was reserved for final evaluation.

### 2.5 ML Model and Interpretabilitys

We chose a RF algorithm for predictive modeling. RF is an ensemble tree‐based method known for its robust performance on classification tasks and its ability to handle both numerical and categorical features without extensive tuning (Tian et al. 2023). It also naturally provides a measure of feature importance.

To enhance the interpretability of the RF model, we employed SHAP (SHapley Additive exPlanations) analysis (Lundberg and Lee 2017). SHAP, a game theory‐based framework, assigns importance values to each feature, elucidating their contribution to predictions at both global (overall feature importance) and individual patient levels for HF versus non‐HF outcomes. Additionally, we applied the Boruta algorithm, a wrapper method built on RF, which iteratively compares real features against random shadow features to identify statistically significant predictors. Boruta confirmed the key features identified by RF's internal importance ranking. Together, RF feature importance, Boruta, and SHAP values provide robust interpretability, mitigating the “black‐box” nature of ML models.

### 2.6 Statistical Analyses

All statistical analyses and calculations were performed using R software (version 4.4.2), *p* < 0.05 was considered statistically significant, and all *p‐values* were two‐sided. Categorical variables were expressed as frequencies and percentages, and comparisons between two groups were made using the chi‐square test or Fisher's exact probability method. Measures that conformed to a normal distribution were expressed as mean ± SD, and independent *t*‐tests were used for comparisons between groups; measures that were not normally distributed were described by the median (upper and lower quartiles) [M(P25, P75)], and nonparametric tests were used for comparisons between groups. For model evaluation, we calculated the following performance metrics on the test set: Accuracy, Sensitivity, Specificity, Precision, F1‐score, and the area under the receiver operating characteristic curve (AUC). The ROC curve was plotted for the RF model's probability predictions, and AUC provides a threshold‐independent measure of discrimination. We also generated a confusion matrix to visualize the model's classification results on the test set. All model development and evaluation steps were done on the training and test splits as described; no test set data were used to tune the model. We present our results following the TRIPOD guidelines for prediction model development and validation.

## 3 Results

### 3.1 Patient Characteristics

The study sample included 1061 patients in total, of whom 589 (55.5%) were diagnosed with HF and 472 (44.5%) did not have HF. The cohort was predominantly male (637 men, 60%, versus 424 women, 40%). Table 1 summarizes the baseline clinical and ECG‐related characteristics of the participants, stratified by HF status. The results showed that age, resting blood pressure, cholesterol, fasting blood glucose, maximum heart rate, exercise‐induced angina, and ST‐segment depression differed significantly (*p* < 0.05) between HF patients and non‐HF patients.

**TABLE 1:** All predictor variables of the study participants with versus without incident HF.

Characteristic	Non‐HF patients (*N* = 472)	HF patients (*N* = 589)	*p*
Age, years	51 (43, 57)	57 (51, 62)	< 0.001
Sex, *n* (%)			
Male	306 (64.8%)	518 (87.9%)	< 0.001
Female	166 (35.2%)	71 (12.1%)	
RestingBP, mmHg	130 (120, 140)	132 (120, 145)	< 0.001
Cholesterol, mg/dL	226 (197, 265)	217 (0, 265)	< 0.001
MaxHR, bpm	151 (135, 168)	128 (113, 145)	< 0.001
Oldpeak, mm	0.00 (0.00, 0.60)	1.20 (0.00, 2.00)	< 0.001
ChestPainType, *n* (%)			
ATA	178 (37.7%)	34 (5.8%)	< 0.001
NAP	152 (32.2%)	78 (13.2%)	
ASY	115 (24.4%)	452 (76.7%)	
TA	27 (5.7%)	25 (4.2%)	
FastingBS ≥ 120 mg/dL			
No	425 (90.0%)	401 (68.1%)	< 0.001
Yes	47 (10.0%)	188 (31.9%)	
RestingECG, *n* (%)			
Normal	314 (66.5%)	326 (55.3%)	< 0.001
ST	65 (13.8%)	130 (22.1%)	
LVH	93 (19.7%)	133 (22.6%)	
ExerciseAngina, *n* (%)			
No	413 (87.5%)	232 (39.4%)	< 0.001
Yes	59 (12.5%)	357 (60.6%)	
ST_Slope, *n* (%)			
Up	356 (75.4%)	92 (15.6%)	< 0.001
Flat	98 (20.8%)	438 (74.4%)	
Down	18 (3.8%)	59 (10.0%)	

Abbreviations: ASY, asymptomatic; ATA, atypical angina; ExerciseAngina, exercise‐induced angina; FastingBS, fasting blood sugar level; LVH, left ventricular hypertrophy; MaxHR, maximum heart rate (bpm) during exercise; NAP, non‐anginal pain; Oldpeak, ST depression induced by exercise relative to rest (mm); RestingBP, resting blood pressure (mmHg); RestingECG, resting electrocardiographic results; ST, ST‐T wave abnormality; ST_Slope, Slope of the ST segment during peak exercise; TA, typical angina.

### 3.2 Model Construction

We randomly divided the dataset into training and validation sets in a 7:3 ratio. Using the training set (742 patients), we built a RF classification model to predict HF. Prior to training, the continuous features in the training data were normalized (Z‐score transformation), and the same scaling was applied to the test data as described in the Methods. The RF model was trained with 100 decision trees; given the relatively modest size of the dataset, this number of trees was sufficient to stabilize the model's performance. We ensured that the training process used only the training subset—the test set remained completely unseen during model training and tuning. Feature selection was implicitly performed by the RF and explicitly examined with the Boruta algorithm on the training set, which confirmed that most features in our dataset had some importance in predicting HF. No features were eliminated from the model, as even those with lower importance were retained to preserve clinical interpretability. After training the RF, we evaluated its performance on the held‐out test set of 319 patients.

### 3.3 Model Performance

On the independent test set, the RF model demonstrated high performance for HF prediction. The AUC was 0.969 on the test set, indicating excellent discriminative ability. In addition to the AUC, Table 2 summarizes the model's other performance metrics on the test data. These metrics collectively show that the RF model achieved a strong balance between sensitivity and specificity, with a slight tilt toward ensuring HF patients are not missed (high sensitivity) at the cost of a few false positives.

**TABLE 2:** Performance of model for prediction.

Model	Specificity	Sensitivity	Accuracy	Recall	F1 score	AUC
RF	0.894	0.938	0.918	0.938	0.927	0.969

Abbreviations: AUC, area under the curve; RF, random forest.

#### 3.3.1 Confusion Matrix

**FIGURE 1:**
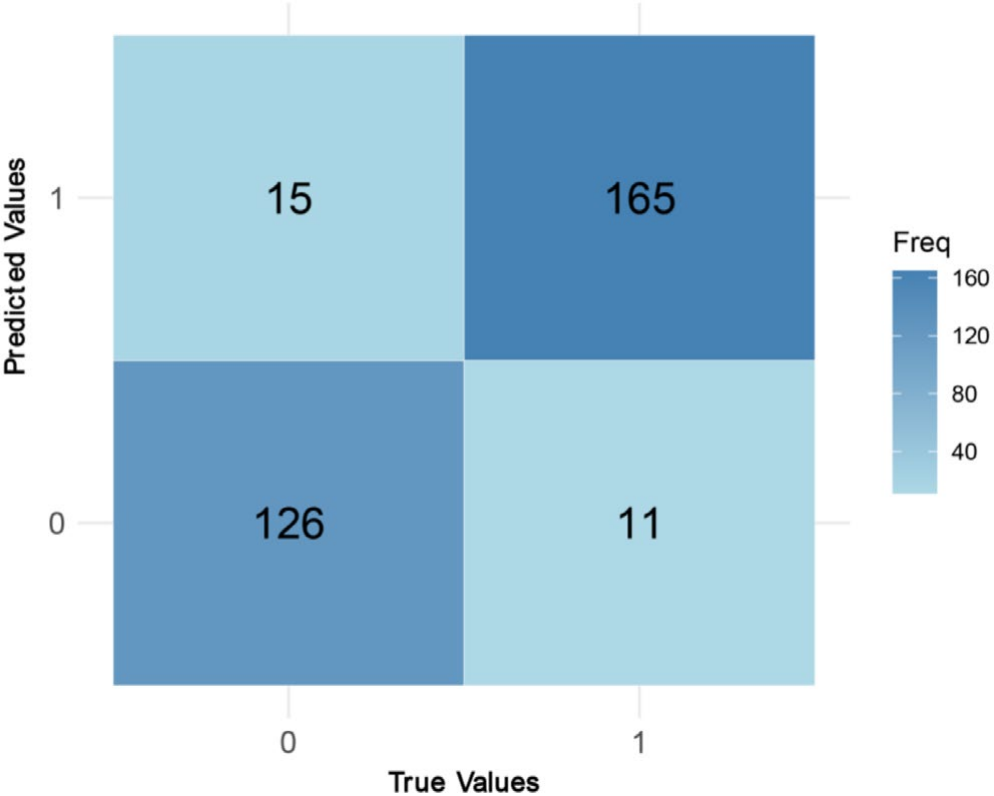
Heatmap of the confusion matrix for the HF prediction model. The intensity indicates the count of patients in each category (darker = higher count). The model shows strong performance, with a majority of patients falling into the true positive or true negative cells and relatively few in the misclassified cells.

To further illustrate the model's performance, Table 3 shows the confusion matrix of predictions on the test set, and Figure 1 visualizes it as a heatmap. The model correctly identified 165 of 176 HF patients and 126 of 143 non‐HF patients, missing 11 HF and misclassifying 15 non‐HF. It has a low false‐negative rate and a moderate false‐positive rate, with its high sensitivity ideal for capturing most HF cases in clinical settings.

**TABLE 3:** Confusion matrix of the prediction model.

	Actual non‐HF patients	Actual HF patients
Predicted HF patients	15	165
Predicted non‐HF patients	126	11

#### 3.3.2 ROC Curve

**FIGURE 2:**
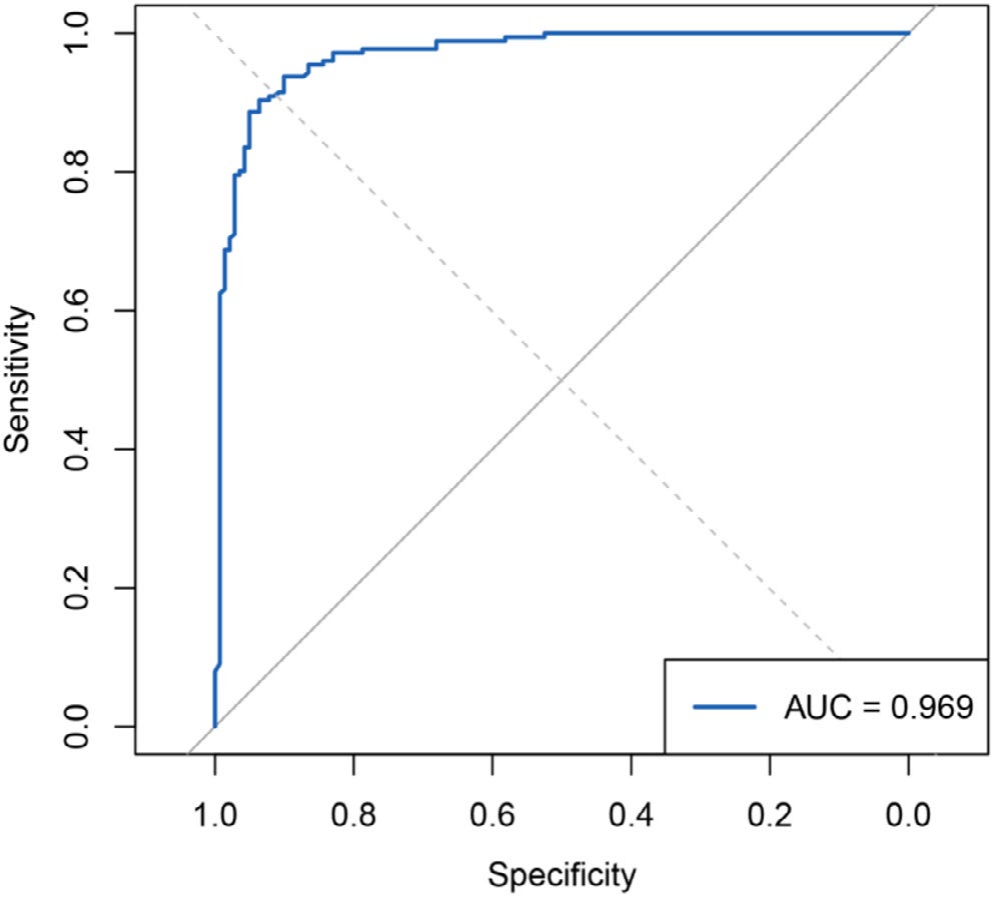
ROC curve of the prediction model.

The following figure demonstrates the ROC curve of the RF model with an AUC value of 0.969 (Figure 2), which indicates that the model has a high discriminatory power.

### 3.4 Feature Importance

**FIGURE 3:**
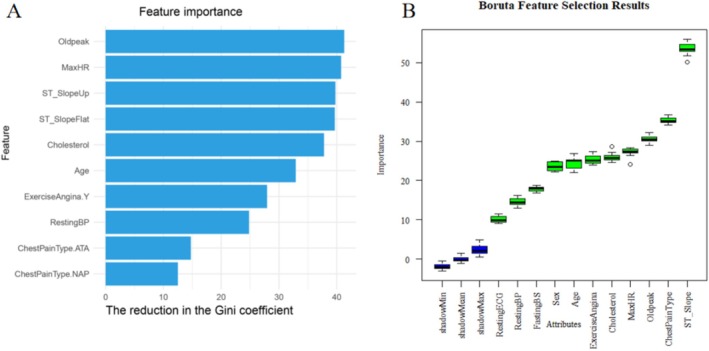
(A) Feature importance plot of the random forest model. (B) Feature importance plot of the Boruta method.

The results of the feature importance analysis using the RF model are shown in Figure 3A. The variable importance plot lists the most important variables in descending order. oldpeak, MaxHR, ST‐Slope, cholesterol level are the most important predictors. Also, we used Boruta's feature selection method and the results are shown in Figure 3B. The green color indicates that the selected elements are important and the blue boxes indicate shading. Each box‐and‐line plot has minimum, average, and maximum importance. Boruta's feature selection method listed ST‐Slope, ChestPainType, Oldpeak, MaxHR, and listed as score as the most important features.

### 3.5 Model Interpretation

**FIGURE 4:**
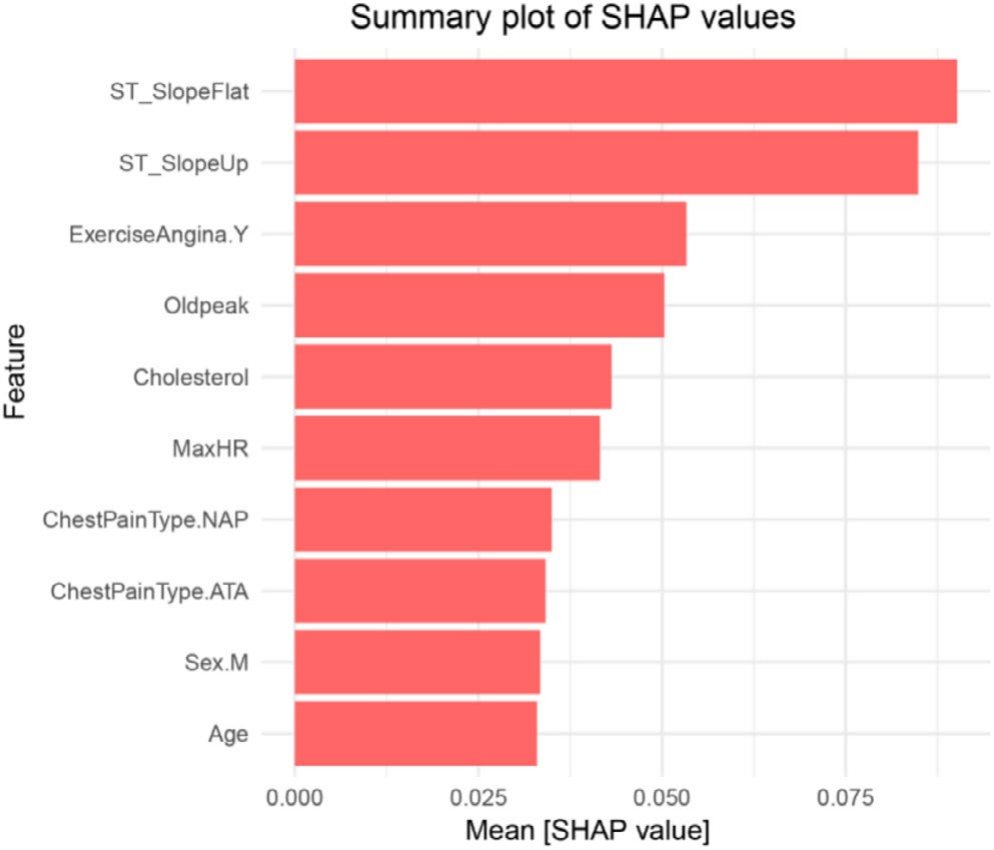
Summary plot of SHAP values.

In order to further understand the decision‐making process of the model, the RF model was interpreted in this study using the SHAP method. The results showed that ST‐Slope, presence of exercise‐induced angina, Oldpeak, and cholesterol level contributed the most to the model's predictions, as shown in Figure 4.

## 4 Discussion

In this study, we developed and validated a RF model to predict the occurrence of HF using data from a public database. A total of 1061 individuals were included, of whom 589 patients (55.5% of the total) developed HF. We used RF and Boruta feature selection methods for characterization. We used the SHAP method to interpret the RF model, which ensured the performance and clinical interpretability of the model. In addition, the performance metrics to assess our model all performed well.

ECG is a widely used, cost‐effective, and non‐invasive tool for evaluating cardiovascular conditions. Although ECG alone cannot definitively diagnose HF, prior research has shown that specific ECG features correlate strongly with HF risk (Hendry et al. 2016). For example, HR measured by ECG closely aligns with 24‐hour ambulatory ECG monitoring results (Camazzola et al. 2024).

Our characteristic importance analysis identified Oldpeak, MaxHR, ST‐Slope, and cholesterol level as the most important predictors. ST‐segment abnormalities on the ECG reflect abnormal ventricular repolarisation, and ST‐segment depression is considered a marker of myocardial ischaemia. Population‐based studies have shown that ST‐segment depression is associated with increased cardiovascular mortality (Larsen et al. 2002; Hedblad et al. 1989). Other studies have shown that ST‐segment depression is independently associated with the prevalence of atrial fibrillation on the 24‐hour ECG monitoring (Johnson et al. 2018). Furthermore, Zègre‐Hemsey et al. (2021) reported that ST‐depression is an independent predictor of new onset HF within 30 days of initial emergency department presentation.

In our study, MaxHR was identified as an indicator with significant predictive value, suggesting that lower maximal HR is significantly associated with the development of HF. Elevated resting HR is thought to be associated with an increased risk of cardiovascular events (Dobre et al. 2014). HR is an important prognostic marker in cardiovascular disease, and lower HR is thought to reduce the incidence of HF and coronary artery disease (Cook et al. 2006; Villacorta 2024; Yip et al. 2016).

ST segment changes may reflect underlying electrophysiological disturbances. Recent studies have shown that ST segment changes play a key role in prognostic assessment in patients with HF. It has been noted that downward‐sloping ST segments are associated with increased all‐cause mortality in both sexes in lateral leads, including lead V5, irrespective of the level of the ST segment (stolahti et al. 2020).

Our study also found that serum cholesterol levels play a significant role in predicting the onset of HF. Low cholesterol levels have been found to be a strong predictor of mortality in patients with ischaemic HF in relatively young adults (Charach et al. 2023). Furthermore, an association between low cholesterol levels and poorer survival outcomes has also been observed in elderly patients as well (Tikhonoff et al. 2005).

The accuracy of our study in predicting HF using 12 variables is higher than that of previous studies (Ramesh et al. 2022; Bhatt et al. 2023). However, our model's AUC of 0.969 is slightly lower than some reported performances. For instance, Sabouri et al. (2023) achieved an AUC of 0.975 using an SVM model. This difference may stem from our focus on ECG indicators, whereas Sabouri et al. (2023) incorporated additional clinical variables (e.g., comorbidities). However, our use of SHAP enhances interpretability‐a critical advantage for clinical adoption‐potentially offsetting the slight performance gap.

The results of this study have important implications for clinical practice. Firstly, by identifying key ECG monitoring indicators, clinicians can identify patients with high‐risk HF earlier, and thus optimize treatment plans and improve patient prognosis. In particular, the monitoring of maximal heart rate and ST‐segment depression can be used as an important reference basis for clinical decision‐making. Secondly, the application of ML models demonstrates its advantages in dealing with complex and variable amounts of medical data. Compared with traditional statistical methods, ML is able to capture the nonlinear relationship between features more effectively and improve the accuracy and reliability of prediction. This provides a new way for individualized treatment of HF and precision medicine.

Our study has several limitations. First, the dataset, sourced from a single Kaggle repository combining multiple heart study cohorts (*n* = 1061), may limit generalizability due to underrepresentation of diverse patient groups (e.g., those with pacemakers or bundle branch blocks). External validation across multicenter, diverse cohorts is needed. Second, the modest sample size constrained exploration of complex models (e.g., deep neural networks) due to overfitting risks and limited subgroup analyses, particularly for women (71 with HF). Larger datasets would enhance model stability and risk stratification. Third, the absence of key comorbidity data (e.g., hypertension, diabetes, obesity) likely reduced predictive power and interpretability, forcing reliance on surrogate features. Including such variables would improve clinical relevance. Fourth, feature importance results, such as the unexpected role of low cholesterol, may reflect dataset‐specific artifacts, warranting cautious interpretation. A parsimonious model could reduce noise. Finally, despite SHAP analysis, the RF model's complexity limits transparency compared to simpler methods like logistic regression. Developing a simplified risk score could enhance clinical adoption. Future work should prioritize broader validation, larger datasets, and integration of clinical variables to improve applicability.

Based on the findings of this study, future research can be conducted in the following areas: multicenter data integration: constructing larger and more diverse datasets by integrating data from different hospitals and regions to enhance the generalization ability and applicability of the model. Introducing more clinical variables: combining more clinical variables such as lifestyle, genetic information, and drug treatment to construct a more comprehensive prediction model and further improve the prediction accuracy. Model optimisation and interpretation: exploring more advanced ML algorithms, such as deep learning models, and combining them with interpretable techniques, such as visualization tools and interpretive algorithms, to enhance the interpretability and clinical application value of the models. Real‐time monitoring and prediction: developing dynamic prediction models based on real‐time ECG monitoring data to help clinicians continuously assess cardiac risk during hospitalization and adjust treatment strategies in a timely manner. Clinical Trial Validation: validating the validity and feasibility of the model through prospective clinical trials to ensure its effectiveness and safety in the actual clinical environment.

## 5 Conclusion

This study successfully developed a predictive model for HF using ECG monitoring indicators and ML methods. Additionally, interpretable ML techniques were employed to identify key risk factors for HF. These findings provide robust data support for clinical practice and help to identify high‐risk HF patients at an early stage and optimize treatment options. However, the study still needs further validation and optimization based on multicenter and diverse data to enhance the value of the model for a wide range of applications.

## Author Contributions

Jia Liu performed the majority of the data analysis, designed the model, and wrote the initial manuscript draft. Dan Zhu contributed to the statistical analysis and helped in refining the methodology section. Lingzhi Deng assisted in data preprocessing, model evaluation, and interpreting the results. Xiaoliang Chen supervised the project, provided guidance on the study design, and reviewed the manuscript.

## Ethics Statement

This study used publicly available, de‐identified data from the Kaggle Machine Learning Repository (https://www.kaggle.com/datasets/rahulrajpvduk/heart‐failure‐prediction‐dataset), and the use of the data adheres to the terms and conditions of the Kaggle platform. As no human subjects were directly involved and no identifiable private information was used, institutional ethics approval was not required.

## Consent

As this was a secondary analysis of a public, anonymized dataset, no informed consent from individual participants was required.

## Conflicts of Interest

The authors declare no conflicts of interest.

## Data Availability

The data used in this study is available from the Kaggle Machine Learning Repository (https://www.kaggle.com/datasets/rahulrajpvduk/heart‐failure‐prediction‐dataset). The source code for the model and data processing is available upon reasonable request.
